# What are the autism research priorities of autistic adults in Scotland?

**DOI:** 10.1177/13623613231222656

**Published:** 2024-02-04

**Authors:** Eilidh Cage, Catherine J Crompton, Sarah Dantas, Khiah Strachan, Rachel Birch, Mark Robinson, Stasa Morgan-Appel, Charlie MacKenzie-Nash, Aaron Gallagher, Monique Botha

**Affiliations:** 1 University of Stirling, UK; 2 The University of Edinburgh, UK; 3 Striving to Transform Autism Research Together – Scotland (STARTS) Network, UK

**Keywords:** adults, policy

## Abstract

Studies investigating autistic community research priorities indicate a mismatch between what autism research focuses on and what autistic people want to see researched. Furthermore, there has not been a research priority-setting exercise specifically with autistic people in Scotland, where there are unique cultural, political and social contexts. Using a community-based participatory design, we aimed to identify the research priorities of autistic adults living in Scotland. Autistic and non-autistic researchers designed and conducted a survey where 225 autistic adults rated and ranked research topics in order of importance and provided qualitative feedback on issues and questions important to them. The top five research priorities were mental health/well-being, identification and diagnosis of autistic people, support services, knowledge and attitudes towards autistic people and issues impacting autistic women. There were differences in priorities according to different intersections of identity, and qualitative responses indicated a desire for research to focus on support and understanding. The bottom three priorities concerned genetics, treatments and interventions and causes. These findings emphasise the need to address the gap between what autism research focuses on and the everyday lives of autistic people.

**Lay abstract:**

Although research has the potential to improve autistic people’s lives, lots of funding goes towards research looking at topics which autistic people say has little impact in their everyday lives. Autistic people’s lives can be different depending on where they live, and Scotland is a unique country in many ways. We wanted to find out which topics autistic people in Scotland want to see research on. Our team of autistic and non-autistic researchers (including university-based and community researchers) created a survey where 225 autistic adults rated and ranked the importance of possible research topics and shared their thoughts on what topics mattered to them. The five most important topics were mental health and well-being, identifying and diagnosing autistic people, support services (including healthcare and social care), non-autistic people’s knowledge and attitudes and issues impacting autistic women. The three least important topics were genetics or biological aspects of autism, autism treatments/interventions and causes of autism. Our findings indicate that autistic people in Scotland want research to focus on things that matter to their day-to-day lives. Also, the Scottish government says they will be listening to autistic people in their latest policy plans, and we believe that considering autistic people’s research priorities is an important part of this. Our findings also add to growing calls for change to happen in how and what autism researchers do research on.

## Introduction

Autism research has the potential to be instrumental in autistic people’s lives, by helping us better understand, support and appreciate autistic people across the lifespan. However, there is also potential for harm caused by autism research, including perpetuating stereotypes and damaging rhetoric which can limit autistic people’s rights (Botha, 2021). Popular areas of autism research are somewhat demonstrated by how autism research is funded. Historically, for example, in the United States, funding between 2017 and 2019 focused on biological research (32.6%) and treatments and interventions (22.9%), with only 5.02% on services and 2.51% on lifespan issues (Harris et al., 2021). In the United Kingdom, funding between 2007 and 2011 mostly went towards studies focused on biology, brain and cognition (56%), with 18% on treatments and interventions, 15% on causes, 5% on services and 1% on societal issues (E. Pellicano et al., 2014). Analysis of Australian autism research funding identified that 27% focused on biological research and 20% on treatments and interventions between 2013 and 2017 (den Houting & Pellicano, 2019). However, den Houting and Pellicano (2019) noted that compared with 2008–2012, increased funding went towards research on services and lifespan issues, although biological research remained dominant. This trend is also shown in the autism research which the National Institute for Health (NIH) funds with only 9% of funding between 2008 and 2018 going on services research with no growth in the proportion (Cervantes et al., 2021). In the same period, prevention research grew significantly from 3.9% to 18%, while roughly 50% consistently went on treatments. Similar patterns are seen in publications: Although there has been consistent growth in the rate of publications (Kirby & McDonald, 2021), molecular genetics constitutes the majority of autism research (Sweileh et al., 2016). Given a lack of consistent methods for tracking funding allocation in autism research globally, we can only rely on data published over differing periods of time. Priorities may have shifted from some of the earlier patterns. Despite this, understanding these historical patterns, including across borders, can still give us keen insight into whether research has met the priorities of autistic people.

However, there has been an increase in participatory research whereby researchers work hand-in-hand with autistic people to do research (e.g. Nicolaidis et al., 2019; E. Pellicano et al., 2022). This approach aligns with a neurodiversity-focused paradigm, which puts autistic people at the heart of research (E. Pellicano & den Houting, 2022). The contemporary research landscape has been shaped by autistic advocacy bringing ‘new ethical, theoretical and ideological debates within autism theory, research and practice’ (Leadbitter et al., 2021, p. 1). This shift has been described as no longer optional, with some funding bodies asking researchers to commit to the meaningful involvement of the people or communities who are typically ‘the researched’ when applying for funding (Pickard et al., 2022).

Despite these moves, most autism research is not focused on the priorities of autistic people. In a systematic review, Roche et al. (2021) identified seven published studies looking at research priorities of autistic people and the wider autism community (e.g. parents, carers, practitioners, researchers). Roche et al. (2021) note that most studies did not use a participatory approach in designing or running the priority-setting exercise. In addition, across the seven studies, only 9% of participants were autistic adults, meaning mostly non-autistic people’s views were represented. For example, in their U.K.-based study, E. Pellicano et al. (2014) asked participants to rate 13 specific research questions (e.g. ‘how can we better recognise signs and symptoms of autism?’). The 13 questions were based on six research areas, including diagnosis, services, societal issues, causes, biology/brain/cognition and treatments and interventions (L. Pellicano et al., 2013). The participants were mostly parents/carers (*n* = 825) and practitioners (*n* = 426), with only 122 autistic adults (7% of sample). The top three priorities of these autistic adults were improving public services, improving life skills and understanding what the future holds for autistic adults. Similarly, a non-peer-reviewed priority-setting exercise by the U.K. charity ‘Autistica’ (Cusack & Sterry, 2017) explored the top priorities of autistic people, their families and professionals (but did not report results according to group), noting interventions for mental health difficulties as the top priority, followed by interventions for the development of language skills and social care support for autistic adults.

Studies outside of the United Kingdom have identified similar priorities. For example, Frazier et al. (2018) conducted a survey completed mostly by those in the United States (86.4%) and by family members (*n* = 4440) rather than autistic people (*n* = 485%–8.1% of sample). Their survey was affiliated with the charity ‘Autism Speaks’, and they asked participants to rate 17 research topics, the majority centred around biological areas such as biomarkers, animal models, genetics or interventions, with only three social issues. Despite the biological bias, the most important topics for autistic adults were the social issues – health and well-being, adult transitions and lifespan issues. Other studies have focused on specific topic areas – for example, priorities within residential care for older autistic adults (Crompton et al., 2020), employment and transitions (Nicholas et al., 2017; Shattuck et al., 2018), mental health (Benevides et al., 2020; Vasa et al., 2018) and sexuality and intimate relationships (Dewinter et al., 2020). Within all these studies, the theme tends to be on priorities with real-world implications for autistic people.

Our study specifically examined the research priorities of autistic people in Scotland. Scotland is in northwest Europe and is currently part of the United Kingdom. However, Scotland is a standalone country with its own diverse geography, culture, politics and people. With a population of around 5,466,000 (Office for National Statistics, 2021), over 80% live in urban areas, even though this constitutes only 2% of the land mass of Scotland (Scottish Government, 2015). Scotland has the lowest life expectancy compared with the other U.K. countries, with significant disparities between the most and least deprived areas (National Records of Scotland, 2021a). In 1999, a new Scottish Parliament opened with devolved powers from the U.K. Westminster government. Devolved powers include health and social care, education, housing, law and local government, among others. The national priorities set by the current Scottish Government focus on improving health and social care, tackling climate change, economic transformation and promoting equality and fairness across society (Scottish Government, 2021c).

Approximately 1% of the Scottish population are estimated to be autistic (Scottish Government, 2018). The Scottish Government (2011) developed the Scottish Autism Strategy where they set out a 10-year plan (2011–2021) to address the challenges faced by autistic people in Scotland and to improve the inclusion of autistic people in Scottish society. Achievements during this period included funding a national postdiagnostic support programme, an independent review of how the Scottish Mental Health Act impacts autistic people, and establishing a national public campaign to promote autism acceptance (Scottish Government, 2021a). However, a review of the strategy identified that progress in the 10 years had been unsatisfactory with ‘limited impact’ and ‘the host of activities and projects had not led to real change’ (Scottish Government, 2021a, p. vii). Since the Strategy ended, the Scottish Government (2021b) published a 2-year ‘Learning/Intellectual Disability and Autism Towards Transformation Plan’. The Plan specifically states that autistic people’s voices will be integral to their work. Given the specific social, political, and cultural context in Scotland, an appreciation of the research priorities of autistic people would be of significant value for informing future Scottish policy and autism research. More generally, conducting research priority studies can help researchers and funders broadly gain insight into the current opinions of autistic people on what research matters to them.

This study thus aimed to identify the research priorities of autistic adults living in Scotland. We used participatory methods, with a team of autistic and non-autistic academic and non-academic researchers working together to design and run a survey. To the best of our knowledge, this is the first fully community-based participatory research priority-setting exercise (Roche et al., 2021).

## Methods

### Community involvement

This study is part of an initiative called Striving to Transform Autism Research Together – Scotland (STARTS), modelled on other community-based participatory research groups such as the Academic Autism Spectrum Partnership in Research and Education (AASPIRE; Nicolaidis et al., 2019). STARTS is a funded network consisting of autistic and non-autistic academic researchers (based at the Universities of Stirling and Edinburgh) and autistic community co-researchers. Five autistic community co-researchers based across Scotland are involved in all aspects of research. They are paid for their time following National Institute for Health and Care Research (NIHR, 2022) Involve guidelines. As a group we meet every month, and in between meetings use email and shared online documents to input ideas and provide feedback. One of the aims of STARTS is to identify the research priorities of autistic people in Scotland. Together, we developed, conducted, analysed and wrote up the current study.

### Participants

We recruited participants by posting adverts on Twitter, Reddit, Discord and Facebook groups, contacting Scottish autistic-led organisations, autism charities, One Stop Shops (support groups for autistic adults), personal contacts, mailing lists consenting to be contacted about research, university disability services and social care organisations working with autistic adults across Scotland. We offered to send these organisations hard copies of the survey with freepost envelopes so that the survey could be completed with supported autistic people (no organisation took this opportunity). We collected data in May to June 2022 and the survey took around 20 min to complete.

In total, 225 autistic adults took part. The majority reported they had a formal autism diagnosis (*n* = 159, 70.7%), 23 were currently seeking a diagnosis (10.2%), 12 were self-identifying but not seeking a diagnosis (5.3%) and 31 preferred not to answer (13.7%). We included all responses to avoid gatekeeping based on diagnostic status, given barriers to diagnosis (Huang et al., 2020). Our survey was available in multiple formats: a standard online version, an online easy-read version (both presented via the survey software Qualtrics) and a downloadable Word document easy-read version (which could be emailed or printed and posted). One hundred seventy-eight participants completed the standard online version, 45 completed the easy read and two completed the Word version. The easy read contained the same content as the standard version but included additional symbols/pictures and simplified language. We also included an option that supporters could help an autistic person complete the survey – six reported they were helping someone and confirmed that all answers represented the views of the person they were supporting rather than their own views.

Participants could skip demographic questions; therefore, the number who responded is reported for each characteristic. The mean age (*n* = 185) was 36.97 (*SD* = 11.58) with a range from 18 to 72. Participants could write their own terms when asked what their gender or sexual orientation was – most reported their gender was female (46.2%) and their sexual orientation was heterosexual (28.4%) or bisexual (15.1%), and most were educated to degree level and in employment (see Table 1). In terms of ethnicity (*n* = 194), the majority reported they were White Scottish, English, Northern Irish or Welsh (*n* = 159, 70.7%). Twenty-five participants reported any other White background (11.1%). Four participants reported mixed or multiple ethnic backgrounds (1.7%), and one participant each reported that they were Black (0.4%), Chinese (0.4%), Pakistani (0.4%) or Latin American (0.4%).

**Table 1.** Participants’ self-reported gender, sexual orientation, highest education level and employment status.

	*N*	%
Gender (*n* = 182)		
Female	104	46.2
Male	38	16.8
Non-binary	24	10.7
Female non-binary	3	1.3
Transgender man	2	0.9
Trans masculine	2	0.9
Genderqueer	2	0.9
Don’t know	2	0.4
Transgender woman	1	0.4
Agender	1	0.4
Non-binary trans-masculine	1	0.4
Quoigender	1	0.4
Autigender	1	0.4
*No response*	*43*	*19.1*
Sexual orientation (*n* = 162)		
Heterosexual/straight	64	28.4
Bisexual	34	15.1
Queer	12	5.3
Pansexual	11	4.9
Lesbian or gay	10	4.4
Asexual	8	3.6
Demisexual	4	1.8
Other/multiple terms	14	6.2
Do not know/ do not care	5	2.2
* No response*	*63*	*28.0*
Highest level of education (*n* = 195)		
None	3	1.3
GCSEs / Standard Grades / National 4 or 5	13	5.8
A-Levels / Highers	22	9.8
National Vocational Qualification	23	10.2
Undergraduate degree	63	28
Postgraduate degree (Masters, Diploma or equivalent)	51	22.7
PhD or other doctoral level qualification	19	8.4
Other	1	0.4
*No response*	*30*	*13.3*
Employment status (*n* = 195, participants could select more than one option)		
Employed full-time	52	23.1
Employed part-time	45	20.0
Self-employed	24	10.7
Unemployed	30	13.3
Retired	5	2.2
Student	36	16.0
Unable to work	29	12.9
Carer	17	7.6
Volunteer	6	2.7
Other	11	4.9
*No response*	*30*	*13.3*

*Note.* GCSE = General Certificate of Secondary Education.

**Figure 1.**
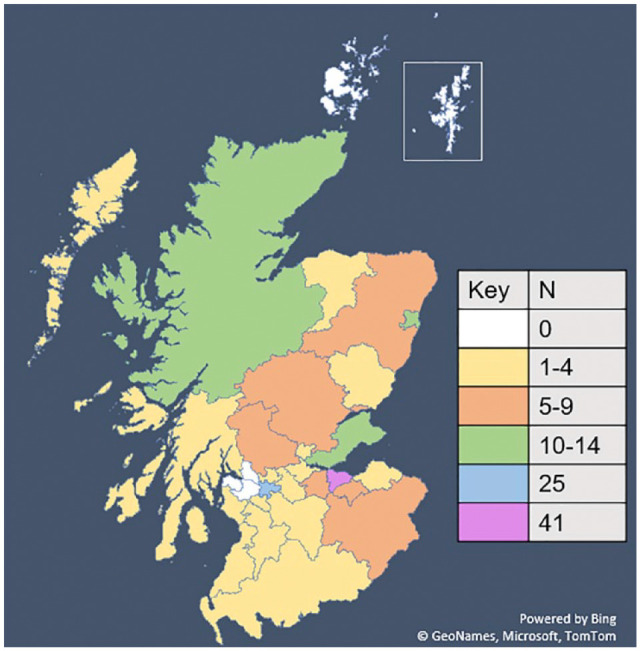
Map of Scotland showing the areas where participants were from (organised by council area).

Participants came from almost all council areas of Scotland (*n* = 192; Figure 1), with most from the cities of Edinburgh (*n* = 41), Glasgow (*n* = 25) and Aberdeen (*n* = 11), with the other most reported areas being Fife (*n* = 11) and Highland (*n* = 11). The only areas not represented were Orkney, Shetland, Inverclyde, Renfrewshire and West Dunbartonshire.

We asked participants whether they were working class (*n* = 188) – 105 said yes and 83 said no; whether they were parents to autistic children (*n* = 192) – 50 said yes and 143 said no; or whether they had any additional disabilities (*n* = 194) – 118 said yes and 76 said no. Here, participants could optionally self-report disabilities, and those who decided to share details most often reported attention-deficit/hyperactivity disorder (ADHD) (*n* = 46), anxiety (*n* = 22), depression (*n* = 20), dyspraxia (*n* = 13), posttraumatic stress disorder (PTSD) (*n* = 12) or Ehlers–Danlos Syndrome (*n* = 9).

We obtained ethical approval from the University of Stirling General University Ethics Panel (GUEP 2022 6978 5962). All participants gave informed consent before starting the survey.

### Materials and procedure

After consent, participants confirmed they were over the age of 18, autistic, and currently living in Scotland. We then showed participants 25 potential research topics (see Table 2), which we had generated in several ways. In a team meeting, autistic co-researchers contributed suggestions for topics into an online brainstorming board, writing down general topics (rather than specific research questions) they thought were both important and unimportant to them and other autistic people. We also reviewed the existing research priorities literature (e.g. Roche et al., 2021) for previously used topics and cross-checked the topics co-researchers had generated against these. The whole group then reviewed the list of topics in a shared document and made suggestions for additions and phrasing to ensure topics were clear. In the survey, participants rated the 25 research topics on a 5-point Likert-type scale from *not at all important* (1) to *very important* (5). After rating each topic, we asked participants to rank the topics in order of importance from 1 (most important) to 25 (least important). We collected both rank and importance ratings so that it would be clear whether topics which fell below the top 10 were still important to autistic, albeit, less so, when made to rank them in comparison with other areas. The higher level of detail can show more generally, what is important, or unimportant to autistic people.

**Table 2.** Descriptive statistics for the research topics, including importance ratings (with 5 being most important), ranking (1 = top priority, 25 = bottom priority) and percentage of participants who put each topic in their top or bottom three priorities.

	Topic	Median rank	% top three	*M* rank (*SD*)	Median importance rating	Mean importance rating (*SD*)	% bottom three
1	Mental health conditions and mental well-being	5.00	40.4	6.79 (5.80)	5.00	4.69 (0.70)	1.8
2	Identifying autistic people/ diagnosis (including diagnostic criteria and postdiagnostic support)	6.00	32.0	8.67 (7.02)	5.00	4.46 (0.89)	3.6
3	Services and supports across the lifespan including social care and healthcare	7.00	26.7	8.36 (6.10)	5.00	4.60 (0.73)	2.2
4	Knowledge and attitudes towards autistic people/ how we view and talk about autism	9.00	21.3	9.92 (6.60)	5.00	4.51 (0.83)	4.0
5	Issues that impact autistic women	9.00	20.0	9.79 (6.16)	5.00	4.41 (0.85)	3.6
6	Employment	10.00	14.7	10.67 (6.44)	5.00	4.44 (0.73)	3.1
7	Interpersonal victimisation and domestic violence/ trauma	10.00	14.7	10.75 (6.14)	5.00	4.44 (0.77)	4.4
8	Education	10.00	12.0	11.04 (6.21)	4.00	4.34 (0.81)	2.7
9	Sensory processing	10.00	10.7	10.73 (6.11)	4.00	4.37 (0.72)	4.0
10	Life skills (including for living independently)	11.00	14.7	11.06 (5.96)	4.00	4.29 (0.80)	1.3
11	Language and communication (including alternative communication methods)	11.00	10.2	11.36 (5.92)	4.00	4.32 (0.79)	1.3
12	Transitions	12.00	7.1	12.37 (5.71)	5.00	4.36 (0.80)	3.1
13	Issues that impact autistic LGBTQIA+ people	13.00	11.6	13.28 (7.46)	4.00	3.96 (1.14)	15.6
14	Friendships and relationships	13.00	8.4	12.12 (5.81)	4.00	4.10 (0.81)	3.6
15	Autistic communication and interaction between autistic people/ autistic community	13.00	6.2	12.86 (6.10)	4.00	4.10 (0.97)	4.9
16	Autistic joy and play	14.00	8.0	13.81 (6.26)	4.00	4.04 (0.91)	6.2
17	Ageing/ older age	14.00	7.1	13.49 (6.63)	4.00	4.12 (0.94)	9.3
18	Physical health conditions	15.00	9.8	13.70 (6.78)	4.00	4.00 (0.92)	4.4
19	Support for family members and understanding family stress	16.00	3.6	15.38 (6.57)	4.00	3.79 (1.02)	14.7
20	Sexual and reproductive health/ sex education	16.00	2.7	15.60 (6.01)	4.00	3.74 (1.09)	13.8
21	Focused interests and/or monotropism	18.00	3.6	16.81 (5.69)	4.00	3.52 (1.05)	11.6
22	Diet and nutrition	19.00	1.30	17.01 (6.19)	3.00	3.44 (1.10)	20.40
23	Genetics or biological aspects of autism, including neuroscience	21.00	6.7	18.02 (6.95)	3.00	3.24 (1.32)	38.2
24	Treatments and interventions (including Applied Behavioural Analysis (ABA), Positive Behaviour Support (PBS), low arousal approach, social skills training, etc.)	24.00	4.0	20.26 (6.56)	2.00	2.47 (1.47)	60.9
25	Environmental and risk factors (causes)	24.00	2.7	21.16 (5.71)	2.00	2.56 (1.33)	66.2

Next, we asked participants to specify what questions researchers should examine within their top three topics only, using an open text response box. In addition, there was an open question asking, ‘Are there any other topics you think should be researched, which we have missed?’ Finally, participants answered demographic questions such as age, gender, ethnicity, sexual orientation and employment status.

### Design and data analysis

We used a mixed-method cross-sectional survey to investigate the research priorities of autistic adults living in Scotland, taking a community-based participatory approach.

We used descriptive statistics to identify the order of priorities. In addition, we identified certain topics may be a higher priority for certain groups within the sample, according to different intersections of identity. We used statistical analyses (*t* tests, correlations) to investigate differences between specific topics, hypothesising that (a) autistic women would rate and rank the topic of ‘issues impacting autistic women’ higher, (b) LGBTQIA+ identifying people would rate and rank the topic of ‘issues impacting LGBTQIA+’ higher, (c) those reporting additional disabilities would rate and rank the topic of ‘physical health conditions’ higher and (d) older participants would rate and rank the topic of ‘ageing/ older age’ higher.

The survey included free-text response questions where participants could provide (a) additional areas of interest within the top 10 topics, and (b) other suggestions for priority topics. We applied conventional content analysis to these data (Hsieh & Shannon, 2005). This is a commonly used qualitative analysis technique and involves coding categories which are directly derived from text data (Hsieh & Shannon, 2005). It is a surface-level method that aims for a simple categorization of small sections of free-text, and not an in-depth or rich exploration of narratives. For our content analyses, three researchers (E.C., R.B., S.D.) analysed the qualitative data, sharing this between them given the volume of data. We first familiarised ourselves with the responses, before identifying potential codes. Codes were then reviewed by these three authors, and categories determined from these codes. Participant responses were then allocated to a category and these categories were then phrased as research questions for the priority areas. The wording of these questions was independently reviewed by a fourth researcher (M.B.) and discussed where necessary with other members of the team until a representative category title was established for each category. After this, another member of the team (M.R.) then independently coded 50% of the raw data for inter-rater reliability, with overall agreement ranging from 60.6% to 100% (median agreement = 84.9%). One researcher (E.C.) checked all disagreements and had a deciding vote on where to allocate codes within categories.

## Results

### Research priority ratings and ranks

Table 2 shows the order of research priorities, with order determined by median rank. Where topics had the same median rank, order was determined by the percentage of participants who put the topic into their top three, and then their mean rank if this information was the same.

### Intersectional analyses

Women were significantly more likely to rank ‘issues that impact autistic women’ higher than everyone else (*t*(146.43) = 2.99, *p* = 0.003, two-sided, Cohen’s *d* = 0.46) with a mean rank of 8.39 (*SD* = 5.52) compared with 11.19 (*SD* = 6.73). They also gave the topic a significantly higher importance rating out of five (*t*(101.47) = –3.90, *p* < 0.001, two-sided, *d* = –0.64), with a mean rating of 4.63 (*SD* = 0.51) compared with 4.10 (*SD* = 1.10). People identifying as LGBTQIA+ were significantly more likely to rank ‘issues that impact autistic LGBTQIA+’ higher than everyone else (*t*(160) = 4.52, *p* *<* 0.001, two-sided, *d* = 0.73) with a mean rank of 10.69 (*SD* = 7.08) compared with 15.89 (*SD* = 7.27). They also rated this topic as significantly more important (*t*(160) = –4.33, *p* < 0.001, two-sided, *d* = –0.70), with a mean rating of 4.32 (*SD* = 0.92) compared with 3.63 (*SD* = 1.12). People who reported additional disabilities did not rank physical health conditions higher than everyone else (*t*(192) = –1.83, *p* = 0.069, two-sided, *d* = –0.27; mean rank 12.27 (*SD* = 6.66) vs. 15.07 (*SD* = 6.69)). However, they did rate physical health conditions as significantly more important (*t*(191) = 3.87, *p* < 0.001, two-sided, *d* = 0.57) with a mean rating of 4.21 (*SD* = 0.79) compared with 3.72 (*SD* = 0.92). There was a significant correlation between participants’ age and ranking ‘ageing/older age’ higher (*r* = –0.39, *p* < 0.001), and between age and importance rating of ‘ageing/older age’ (*r* = 0.30, *p* < 0.001), such that older participants were more likely to rate the topic as more important.

### Qualitative responses

For their top three topics, we asked participants to share their views and ideas on what questions or areas were of importance within these. Below, for the top 10 priorities identified (Table 2), we outline the top three questions within each topic, with *n* indicating the number of comments made, and provide an example quote (all questions identified, and additional supporting quotes for each specific question within each priority, are in Supplementary Material).

*Priority 1: Mental health and mental well-being.* Eighty-four participants provided a response detailing their priorities within this topic. The top three questions were the following:

1. How can mental health services meet the needs of autistic people and what supports are helpful? (*n* = 69)
2. What causes autistic people to experience mental health issues? (*n* = 44)
3. How do we define autistic well-being and what factors contribute to positive autistic well-being? (*n* = 30)

Example quote: ‘They should look into what mental health support actually is helpful for autistic and what modifications can be made to current services’.

*Priority 2: Identifying autistic people / diagnosis.* Seventy participants provided a response discussing their specific priorities. These were the following:

1. How can the diagnostic criteria be improved or redefined to more accurately reflect the true nature of autistic experience, taking into account neurodivergence and intersectionality? (*n* = 67)
2. How do we ensure autistic people and families/carers/partners get access to high quality pre- and postdiagnostic support that is helpful to them? (*n* = 37)
3. What are the barriers and facilitators to accessing a diagnosis and having a positive diagnosis experience, and how can barriers be reduced? (*n* = 26)

Example quote: ‘How can we address the lack of pre- and postdiagnostic support for potentially autistic people, their families and carers?’

*Priority 3: Services and supports across the lifespan.* Sixty-six participants provided a response, with the top questions identified as follows:

1. How can services be designed or adapted to be more person-centred and high quality for autistic people across the lifespan, with intersectional needs and conditions considered? (*n* = 35)
2. How can we increase and/or improve access and information about support services, with choice, autonomy, advocacy and agency prioritised? (*n* = 28)
3. How can we improve understanding of autistic people and their needs among people who work in services? (*n* = 26)

Example quote: ‘The importance of continued support throughout the autistic person’s life, not just focusing on autistic youth’.

*Priority 4: Knowledge and attitudes towards autistic people.* Fifty-two participants responded, with their top questions identified as follows:

1. How do we prevent stigma and increase autism acceptance? (*n* = 29)
2. How can we better understand stigma and prejudice, including its causes and consequences? (*n* = 20)
3. How can we reduce discrimination in specific settings (e.g. at work, in education, police, the media, autism research)? (*n* = 15)

Example quote: ‘How does stigma impact the success of autistic people? How much does it contribute to burnout/suicide rates?’

*Priority 5: Issues impacting autistic women.* Forty participants specified their areas of interest, with the most mentioned questions as follows:

1. What causes autistic women to be less/ mis-diagnosed and how can we make diagnoses/diagnostic criteria and diagnostic support more accessible and adequate for autistic women? (*n* = 41)
2. How can we shift stereotypes of autism and promote knowledge and acceptance of women’s experiences and differences, including other intersections of identity? (*n* = 31)
3. How can we better understand autistic women’s experiences of masking and its impacts (on their lives, mental health and diagnosis)? (*n* = 16)

Example quote: ‘Why are we so bad at diagnosing women and girls?’

*Priority 6: Employment.* Thirty-one participants responded, with the top questions of

1. What workplace support and reasonable adjustments work best for autistic people, to help them do well in the workplace and feel comfortable disclosing being autistic? (*n* = 23)
2. How can workplaces be made more inclusive and less discriminatory, with a greater appreciation of autistic people’s needs? (*n* = 16)
3. What changes are needed to help more autistic people access work, for example, to interviews and applications? (*n* = 14)

Example quote: ‘How can autistic employees be better supported?’

*Priority 7: Interpersonal victimisation, domestic violence and trauma.* Thirty-two participants responded, and we identified the top questions as:

1. What are the causes and risk factors of trauma and victimisation for autistic people? (*n* = 22)
2. Are trauma/victim support services/approaches meeting the needs of autistic people, and what is helpful for recovery/creating safety? (*n* = 20)
3. How can trauma and victimisation be prevented (and recognised early) in autistic people? (*n* = 17)

Example quote: ‘What is the true impact (personally, mentally, socially, economically etc.) of trauma/ interpersonal violence?’

*Priority 8: Education.* Twenty participants responded, and the top questions were the following:

1. How can educational institutions best support autistic individuals to reach their full potential (including into the future)? (*n* = 20)
2. What does the most accessible and inclusive educational environment look like? (*n* = 10)
3. How can we make teachers and other educational professionals more understanding about the needs of autistic individuals? (*n* = 7)

Example quote: ‘How can educational environments be made more accessible?’

*Priority 9: Sensory processing.* Sixteen participants responded, with top questions identified as follows:

1. What are the underlying mechanisms or causes of sensory processing differences and sensory overwhelm? (*n* = 9)
2. How can sensory environments be adapted and improved? (*n* = 8)
3. How do autistic people’s senses work and how is this different from neurotypicals? (*n* = 7)

Example quote: ‘How does autistic sensory processing work differently–structurally and functionally?’

*Priority 10: Life skills.* Twenty-four participants responded, with their top questions as follows:

1. How can autistic people be best supported with the skills needed for living independently and everyday life? (*n* = 24)
2. What support would help autistic people to manage money and their finances? (*n* = 5)
3. How can autistic people be supported to self-advocate and understand their own needs, including recognising burnout? (*n* = 4)

Example quote: ‘There is not enough support to help autistic adults learn and prioritise the life skills they need to live independently’.

### Other topics

When asked whether there were other topics that should be researched, 109 participants responded. Although we asked participants to describe topics we had missed, many wrote about topics that appeared within our 25 topics, which may indicate some used the opportunity to write about topics they thought were important but had not made their top three. We, therefore, moved any comments (62.1% of total comments) pertaining to preexisting topics and analysed them within these topics (e.g. if someone mentioned diagnosis, this was analysed alongside all other comments about diagnosis).

There were 67 remaining comments from 50 participants. Most often, participants mentioned the need to examine co-occurring conditions (*n* = 11), for example, ‘Other disabilities/conditions (EDS, MCAS/allergies) and how those overlap and intersect with autism and all the other various learning differences also common to autistics such as ADHD, dyslexia, etc.’ Next, participants mentioned rejecting applied behavioural analysis (ABA)/‘normalcy’ interventions (*n* = 9), for example, ‘Alternative supports to ABA and other behavioural approaches which are damaging to autistic people as they are trying to train them rather than support and respond to the way the autistic mind works’. Considering intersectionality was also mentioned (*n* = 8): ‘Anything and everything to do with the experiences of autistic people who are NOT white or who are raised in a marginalised culture (whether they be from immigrant families or Scottish Travellers)’. Others discussed research on cognition (thinking and learning; *n* = 5): ‘Challenges and strategies to cope with working/short term memory problems’. Participants also mentioned autistic parenting (*n* = 6) and pregnancy for autistic people (*n* = 5): ‘Not parents of autistic children, but autistic parents themselves, in particular those with autistic children’. Finally, participants highlighted the menopause (*n* = 5): ‘the effect of the menopause on autistic people’. Topics mentioned by four or fewer participants are shown in the Supplementary Material.

## Discussion

The top priorities of autistic people living in Scotland focused on issues with implications for the everyday lives of autistic people across the lifespan. For example, the top five priorities concerned mental health and well-being, identifying and diagnosing autistic people, support services, knowledge and attitudes towards autistic people and issues impacting autistic women. The bottom three priorities were genetics, treatments and interventions and causes. From the questions identified through participants’ qualitative responses, there was a focus on improving support and understanding. We also noted intersectional differences in the priorities of different groups according to other identities which autistic people held. Our findings may not be surprising to many, and yet autism research continues to focus on topics which do not align with these priorities.

Our findings are consistent with prior work on research priorities of autistic people elsewhere (Roche et al., 2021). Previous U.K.-wide studies have differed slightly in the topics rated as priorities: In E. Pellicano et al’.s (2014) study, autistic adults’ top priorities were services, life skills, and understanding what the future holds for autistic adults. In Autistica’s priority-setting exercise (Cusack & Sterry, 2017), the top priorities (not reported for autistic people only) focused on mental health interventions, communication/language interventions and social care services. Our participants’ top priorities similarly prioritised mental health and support services, but also considered diagnosis, knowledge and attitudes, and issues impacting autistic women. Irrespective of the specific topics, the message remains the same: Autistic people want research to have a meaningful impact in their lives.

Although the order of topics is of interest, participants viewed most topics (21 out of 25) positively with a median importance rating of four or five. This shows that while the ranking of topics is important, it cannot be considered in isolation without the importance ratings. Lower ranked topics still had high support from the autistic community (e.g. communication and language research, or transitions research), meaning nuance would be lost if we focused on rank alone. Participants also mentioned several topics which we did not originally list, such as autistic parenting and co-occurring conditions. Furthermore, many topics are intertwined, as indicated in the qualitative responses, which centred around support, improving accessibility, and increasing understanding among non-autistic people. It is, therefore, important not to view topics in isolation. What is clear was that research focused on genetics, treatments, interventions and causes was not a priority – both their rank and importance ratings were low. This was especially the case with behavioural intervention studies, and research on the causes of autism which had low ranks, importance ratings and were in the bottom three in more than 61% and 66% of the sample, respectively (compared with even biological research, which while it was ranked consistent low, was only in the bottom three ranks for 38%). Several participants specifically mentioned that research should explore alternatives to practices such as ABA or positive behaviour support (PBS). Throughout, the questions identified a desire for support – but critically, support that does not seek to *treat* or *intervene* in making someone less autistic.

Ultimately, the contrast between what research is happening and autistic people’s priorities highlights how the research community is failing autistic people. In the United States, $394 million was spent on autism research in 2018 (U.S. Department of Health & Human Services, 2021), while £10.4 million was spent in the United Kingdom in 2016 (Warner et al., 2019). Given such investment, there should be careful consideration of how we prioritise autism research funding. For example, there are substantial research challenges, including addressing high suicide rates (Cassidy & Rodgers, 2017), health disparities (Kinnear et al., 2019; Weir et al., 2022), early mortality (Hirvikoski et al., 2016), disproportionate victimisation (Griffiths et al., 2019) and dehumanisation of autistic people (Cage et al., 2019). Funding bodies should require clear statements of relevance and impact for the everyday lives of autistic people in applications.

Comparing autistic adults’ research priorities with funded research shows the stark contrast between priorities for autism research. In U.K. funding, 27% of funding was spent on the top 10 priorities identified by ‘Autistica’ (Warner et al., 2019), although some of these priorities included interventions as they consulted a wider group (non-autistic parents/caregivers, practitioners). Interventions were not a priority for our solely autistic sample, which means that even less funded research would meet the priorities of autistic adults in Scotland. Furthermore, while our sample was adamant, the most important topics concerned applied science, a review of what is published found that the vast majority of what is published falls under basic science (molecular genetics; Sweileh et al., 2016). We argue that funding which fits with autistic people’s priorities would be more cost-effective in developing services that meet autistic people’s needs, rather than investing funding which fails to meet the everyday needs of autistic people across the lifespan.

Our participants also noted the need for research to attend to intersectionality. Autism research fails to include marginalised groups (Cascio et al., 2021; Giwa Onaiwu, 2020). Intersecting identities are often ignored in autism research, with researchers reducing autistic people down to being autistic alone and failing to consider race or ethnicity, gender, sexuality, socio-economic status or other key factors which may affect experiences (Botha & Gillespie-Lynch, 2022). In a review of autism intervention literature, fewer than 25% of papers collected or reported information on participants’ race (Steinbrenner et al., 2022). Our analyses indicated there will be different priorities for people within different intersections of marginalisation, reiterating that the autistic community is not a homogeneous group, and neither are their research needs. Intersections of marginalised communities within the autistic community should not have their needs homogenised by the wider autistic community who do not share them; otherwise, the most marginalised sections of the autistic community will fail to have their needs recognised and attended to.

Our study has several strengths and limitations. Although there was a good geographical spread of respondents, there were no responses from five council areas, including those with fewer autistic-led organisations. Two of these areas were island populations that may have unique responses we have not gleaned. According to the 2011 Scottish Census, our sample was also more educated than the most qualified council area in Scotland (59.5% in our sample compared with 41.4%; National Records of Scotland, 2021b). Despite the high level of qualification overall, the rate of unemployment in the sample (13.3% being a conservative estimate without potentially accounting for those who are unable to work or carers) is well beyond the national rate (3.1%). Furthermore, the 2011 Scottish Census shows that 96% of Scotland’s population was White, comparable with our sample (National Records of Scotland, 2021c). Nonetheless, our participants identified intersectionality related to race and ethnicity as important. Further research with Black, Asian and Ethnic Minority communities is desperately needed (Malone et al., 2022). Only 2.6% of our sample had additional help from a caregiver or other adult to fill in the survey, meaning that it is likely that the views of some autistic people with a learning disability have not been captured in this survey. This may be reflected in the priority-setting exercise with communication research receiving a lower rank (although its importance rating still puts it relatively high). Importantly, however, nearly 21% of our sample opted for the easy-read version of the survey, so it is possible that the alternative formats available made the survey accessible to more autistic people, therefore potentially facilitating more diversity (although not enough) in our sample. There are interests that are shared among autistic people; however, autistic people with learning disabilities may have needs that are not articulated here or are less represented than they otherwise would have been (such as communication research). Although our sample reflects only the views of 225 autistic people in Scotland, this sample size is larger than many other research priority studies, where often non-autistic people’s views dominate (Roche et al., 2021).

There are clear implications from our research. The Scottish Government’s (2021b) ‘Learning/Intellectual Disability and Autism Towards Transformation Plan’ aims to centre autistic people within initiatives, policy-making and future development plans. Given that a large proportion of U.K. autism research funding has been allocated in Scotland (Warner et al., 2019), Scotland could be a hub for autism research. Yet, we need to ensure it is a progressive hub, aligned with the priorities of autistic people. Our findings should directly influence strategic planning for both research and social policy by providing guidance to the issues most important for autistic people in Scotland. Furthermore, we all need to do more to bridge the gap between research and policy, to ensure Scottish autism research not only focuses on the research areas important to autistic people, but translates this into practicable, implementable policy.

Outside of Scotland, our research adds to repeated calls for change to happen within autism research (e.g. Botha, 2021). Decades of research constrained within a medical model paradigm have restricted what could be possible with autism research (E. Pellicano & den Houting, 2022). We implore autism researchers to listen to autistic people and consider how autism research could make a meaningful difference to autistic people’s everyday lives and to make more effective use of funding. We call on funders to fund traditionally under-researched areas and provide sufficient funds for participatory approaches. We hope that the future of autism research is unified, progressive and impactful.

## Supplemental Material

sj-docx-1-aut-10.1177_13623613231222656 – Supplemental material for What are the autism research priorities of autistic adults in Scotland? Supplemental material, sj-docx-1-aut-10.1177_13623613231222656 for What are the autism research priorities of autistic adults in Scotland? by Eilidh Cage, Catherine J Crompton, Sarah Dantas, Khiah Strachan, Rachel Birch, Mark Robinson, Stasa Morgan-Appel, Charlie MacKenzie-Nash, Aaron Gallagher and Monique Botha in Autism
