# Supplementary material for: What are the autism research priorities of autistic adults in Scotland?
Source: Autism. 2024 Feb 4;28(9):2179–90. doi: 10.1177/13623613231222656 (PMC11401337; doi:10.1177/13623613231222656)
Supplement: sj-docx-1-aut-10.1177_13623613231222656 – Supplemental material for What are the autism research priorities of autistic adults in Scotland? [file sj-docx-1-aut-10.1177_13623613231222656.docx]

**Supplementary material**

1. **All questions identified using content analysis for the top ten topics, with number of comments (n) and supporting example quotes.**

| **Topic and identified questions** | **n** | **Example quotes** |
| --- | --- | --- |
| ***Priority one: Mental health*** | | |
| 1. How can mental health services meet the needs of autistic people and what supports are helpful? | 69 | *“They should look into what mental health support actually is helpful for autistic and what modifications can be made to current services.”*  *“How can you tailor treatment for anxiety (or other MH condition) to someone with autism?”* |
| 1. What causes autistic people to experience mental health issues? | 44 | *“What are the key issues that contribute to poor mental health in autistic people?”*  *“Understanding why autistic people are so vulnerable to various mental health issues”* |
| 1. How do we define autistic wellbeing and what factors contribute to positive autistic wellbeing? | 30 | *“What does autistic wellbeing look like?”*  *“How can we help autistic people find community, activities, hobbies and fulfilment?”* |
| 1. What training and/or experience do professionals need to improve mental health outcomes and experiences for autistic people (e.g., GPs, therapists, psychiatrists, psychologists, social workers etc.) | 25 | *“The sparsity of any decent training in autism for mental health professionals from an actually autistic viewpoint.”*  *“How can mental health team and support organisation to be more flexible, less dogmatic (recycling tired cliches) in their approach to supporting autistics.”* |
| 1. How can mental health issues and suicide be prevented for autistic people? | 20 | *“How can we lower the suicide rate of Autistic people?”*  *“How can we help autistic people avoid common mental health conditions which are prevalent within the autistic population?”* |
| 1. Are autistic people, particularly women, being misdiagnosed with mental health conditions, and if so, how can this be prevented? | 17 | *“Are autistic people being misdiagnosed with mental illnesses such as BPD or bipolar? Anecdotally this seems common - it would be helpful to know to what extent this seems to happen and why”*  *“Appropriate interventions to prevent misdiagnosis of mental health disorders.”* |
| 1. Do mental health issues present differently in autistic people? | 15 | *“The existing symptoms/ diagnoses/ terminology may not reflect those that are autistic when compared to those that aren't.”*  *“I would particularly be interested in how my experience of bipolar is different from a non-autistic person’s experience of the same condition. I'm also particularly fascinated by autistic manifestations of PTSD, as I feel this is often different from the non-autistic presentation.”* |
| 1. What mental health issues do autistic people experience and how common are they? | 8 | *“Are there mental health problems associated particularly with autism and if so why?”*  *“What conditions are autistic people more pre-disposed / vulnerable to?”* |
| ***Priority two: Identifying autistic people / diagnosis*** | | |
| 1. How can the diagnostic criteria be improved or redefined to more accurately reflect the true nature of autistic experience, taking into account neurodivergence and intersectionality? | 67 | *“Diagnosis needs to become a peer-led, supportive process that starts with autistic experience, not "behaviour" or "symptoms".”*  *“Diagnostic criteria MUST be updated to include the many presentations of autism, and practitioners particularly need continued education about non-cis-het-white male presentation. Too many people are getting missed and are struggling so much. There is no 'male' or 'female' presentation, that's bunk. Researchers could look at how to get more cutting edge and gender/queer aware diagnostic tools into clinical practise sooner.”* |
| 1. How do we ensure autistic people and families/ carers/ partners get access to high quality pre- and post-diagnostic support that is helpful to them? | 37 | *“How can we address the lack of pre- and post-diagnostic support for potentially autistic people, their families and carers?”*  *“There is little to no support whatsoever after diagnosis, in particular for adults so this needs to be addressed as well.”* |
| 1. What are the barriers and facilitators to accessing a diagnosis and having a positive diagnosis experience, and how can barriers be reduced? | 26 | *“What are the hurdles that are currently preventing autistic people from getting a diagnosis? Should we be trying to remove those hurdles?”*  *“The difficulty in getting through the [diagnosis] process with the GP. In my personal experience they did not want to diagnose me as I was an adult and they said they didn't want to put a label on me. One of my friends who was diagnosed as an adult as well had a similar issue and it requires us to press them which is difficult.”* |
| 1. What training/ education/ experience do professionals need to improve diagnostic outcomes? | 21 | *“Community health services (inc. GPs) should be TRAINED to look for the signs and traits, instead of just condemning people to a misdiagnosis of depression, BPD, anxiety disorder.”*  *“How can medical professionals better understand the different experiences and expressions of autism?”* |
| 1. What can we do to identify autistic people as early as possible? | 15 | *“Huge numbers of people go undiagnosed and therefore are not offered support, how do we avoid this?”*  *“How can we make sure that autistic females are diagnosed as early as possible”* |
| 1. How long does it take to receive a diagnosis, and how can the speed of the diagnostic process be increased? | 12 | *“How to more accurately and rapidly identify autistic people - whether children or adults”*  *“How long on average it takes an autistic person to be diagnosed with autism”* |
| 1. How can we ensure autistic people without a diagnosis or those misdiagnosed/missed still get the support they need, particularly when experiencing other mental health difficulties? | 11 | *“How many deaths by suicide were because of undiagnosed untreated autism?”*  *“Mis-diagnosis with mental health issues due to missed early correct Autism diagnosis. A new system of looking at what happens to mental health when autism diagnosis is missed.”* |
| ***Priority three: Services and supports across the lifespan*** | | |
| 1. How can services be designed or adapted to be more person-centred and high quality for autistic people across the lifespan, with intersectional needs and conditions considered? | 35 | *“People are likely to need healthcare and social care at times in their lives, whether they are autistic or not. Autistic people may have other health conditions for which they need support. This support has to be available in an autistic-friendly format.”*  *“The importance of continued support throughout the autistic person’s life, not just focusing on autistic youth.”* |
| 1. How can we increase and/or improve access and information about support services, with choice, autonomy, advocacy and agency prioritised? | 28 | *“How can we support autistic people to live independently, access information and help, advice created by other autistic people (not from a NT or psychiatric perspective), and help them access support and advocacy groups”*  *“How can we help autistic people understand what support they are entitled to throughout their lifetime.”* |
| 1. How can we improve understanding of autistic people and their needs amongst people who work in services? | 26 | *“How do we ensure healthcare professionals, social workers and other people who provide support understand autism and are helping autistic people to make the best choices for their health and wellbeing?”*  *“Training for medical staff such as GP / A&E and anyone who triages should be improved.”* |
| 1. How can the accessibility of healthcare be improved, particularly the GP? | 22 | *“Are current booking systems preventing autistic people getting treatment?”*  *“It would be really good to look as accessing healthcare for Autistic people - for example the pain differences, sensory discomforts such as touch sensitives, the lights being too bright, understanding questions and communication differences.”* |
| 1. How can autistic people be better supported to access social care supports and welfare payments? | 14 | *“Regarding social care, there needs urgently to be a complete revision of the benefits system, specifically PIP, so that autistic people are not attempting to force a physical disability oriented system to fit their own experience. This needs research all of its own.”* |
| 1. Are services/supports causing harm and what can be done to prevent this? | 13 | *“How can we identify harmful “supports“ and “services“, how can autistic people get away from them, how can they be reported, the people responsible stopped and prevented from doing further harm, how can autistic people be safe within the supports and services system - especially those of us with various communication difficulties and differences?”*  *“Why are so many autistic people subject to long stay detention in inappropriate mental health hospitals when they should be allowed to thrive in the community?”* |
| ***Priority four: Knowledge and attitudes towards autistic people.*** | | |
| 1. How do we prevent stigma and increase autism acceptance? | 29 | *“How can we reduce the stigma around being autistic?”*  *“I think that still lots of people don't understand autistic people and our needs and it’s frustrating. It would be great to look into people perceptions of autism and how these can be changed.”* |
| 1. How can we better understand stigma and prejudice, including its causes and consequences? | 20 | *“Where the stigma comes from and how it can be addressed at an early age. The greater the awareness of autism at younger ages, the better.”*  *“How does stigma impact the success of autistic people? How much does it contribute to burnout/suicide rates?”* |
| 1. How can we reduce discrimination in specific settings (e.g., at work, in education, the criminal justice system, the media, autism research, etc.)? | 15 | *“Looking at the education and medical profession and what they understand of autism.”*  *“How can research practices and principles in the future be improved to address past stigma and discrimination?”* |
| 1. How are diagnosis, disclosure and stigma inter-related? | 12 | *“How does stigma and misinformation feed into the diagnostic process”*  *“How can we reduce the stigma around being autistic? Should we be changing the way we diagnose autism, as this might be a part of the problem?”* |
| 1. How can we ensure autistic people are heard and listened to when it comes to changing societal attitudes? | 6 | *“The centring of non-autistic family members and how this leads to further stigma and lack of support”*  *“There is a lot of stereotyping of autistic people in society and also not enough chance for autistic adults to advocate for themselves to give others more idea of what autism actually is.”* |
| 1. What can we do about internalised stigma? | 5 | *“How can we make it easier for those who don’t accept their diagnosis of Autism?”*  *“Before I started looking into my own situation I had many misconceptions and stereotypes about autism, I had some really bad moments the closer I got to finding the answer. Like I didn’t want that for myself. I’d like to know how many people went through the same if possible, I’d like for autism to be seen in a better light and to be taught about more to help. I really hope that no one hates themselves because of this reason, I’ve since moved past any feelings like that.”* |
| 1. How can we take into account the role intersectionality plays in stigma? | 3 | *“Benefits of diversity (need for representation of adults, women, BIPOC, and lower support needs autistic people in resources, media, and so on)”* |
| ***Priority five: Issues impacting autistic women.*** | | |
| 1. What causes autistic women to be less / mis-diagnosed and how can we make diagnoses/diagnostic criteria and diagnostic support more accessible and adequate for autistic women? | 41 | *“Understanding why diagnosis is so hard for many women and girls and improving the diagnostic process.”*  *“Why are we so bad at diagnosing women and girls?”* |
| 1. How can we shift stereotypes of autism and promote knowledge and acceptance of women's experiences and differences, including other intersections of identity? | 31 | *“Why do some people still think that girls can't be autistic? Most people are only educated on what autism might look like in a young male child, but not when it comes to autism in girls.”*  *“Realise that girls CAN be autistic and should be supported at the earliest stage possible rather than being dismissed as badly behaved.”* |
| 1. How can we better understand autistic women's experiences of masking and its impacts (on their lives, mental health, and diagnosis)? | 16 | *“Understanding masking and why it is more necessary for autistic women and girls.”*  *“How do we better identify autism in women who are masking?”* |
| 1. What are the issues and factors impacting on autistic women's mental health and how can we improve mental health for autistic women? | 10 | *“Mental health experiences of women: incorrect diagnoses of personality disorders, bipolar disorder, and treatment of mental ill health that is helpful for autistic people”*  *“Specifically, (special interest), emerging research showing big correlation in people with eating disorders are now being diagnosed as autistic (primarily women because 9/10 people with eating disorders - that we know of - are women) - so screening in eating disorder services and looking at what support is needed in eating disorder services”* |
| 1. What support (including healthcare) is available for autistic women, and how can support be improved or designed to meet autistic women's needs? | 9 | *“Autistic women's healthcare and ensuring their experiences are listened to.”*  *“The way in which almost all support for Autistic people and the process for a diagnosis are designed just for cis men.”* |
| 6= How can we better understand and support autistic women's experiences of pregnancy and parenthood? | 8 | *“Difficulties being pregnant and sensory overload while feeding - especially unidentified as autistic at the time”*  *“Menstrual cycle, pregnancy, menopause”* |
| 6= What can we do to support autistic women who are victims of violence / discrimination, and prevent this from happening? | 8 | *“Are autistic women at higher risk of gender-based violence? If so, why, and what can be done to prevent this and support survivors?”*  *“Sexual violence against autistic women.”* |
| 1. What are the physical health issues and conditions experienced by autistic women and their impacts? | 6 | *“Women and AFAB [assigned female at birth] people do appear to have higher rates of some of the co-occurring conditions such as allergies and immune/autoimmune problems so this could do with some examination.”*  *“What can be researched about hormonal changes for autistic women (the sensory differences with this)?”* |
| ***Priority six: Employment*** | | |
| 1. What workplace support and reasonable adjustments work best for autistic people, to help them do well in the workplace and feel comfortable disclosing being autistic? | 23 | *“How can autistic employees be better supported?”*  *“How can employers develop Autistic People who are already in work so that their skills are brought to the fore?”* |
| 1. How can workplaces be made more inclusive and less discriminatory, with a greater appreciation of autistic people's needs? | 16 | *“How does workplace culture and recruitment irrationally bias employers against autistic people”*  *“I see way too many talented autistic adults suffering at their workplaces unnecessarily due to their employers ignorance. Disability inclusion should be on every employers agenda.”* |
| 1. What changes are needed to help more autistic people access work, for example to interviews and applications? | 14 | *“As an autistic person I struggle with all aspects of job hunting and obtaining work. It would be good to research and understand how this could be made easier”*  *“Do recruitment techniques discriminate against autistic people?”* |
| 1. Why are fewer autistic people employed, including what systemic issues disproportionately affect them and how can these issues be tackled? | 8 | *“Why is the percentage of autistic people in work so very low (29% in work, of which only just over half are full-time).”*  *“Why autistic adults are employed at such a low level? Whether this is related to the workplace’s knowledge, interview skills or something else? How can the employment rate be changed?”* |
| 1. In general, what are autistic people's experiences of work? | 5 | *“What do autistic people say are the biggest barriers they face in employment?”*  *“It would be good to collect stories of autistic people's experiences at work, good and bad. And strategies for dealing with problems.”* |
| 1. What is self-employment like for autistic people and how can they be best supported within this? | 3 | *“How autistic people can be supported to be self employed, including handling aspects of their jobs they find more challenging than others.”* |
| ***Priority seven: Interpersonal victimisation, domestic violence and trauma.*** | | |
| 1. What are the causes and risk factors of trauma and victimisation of autistic people? | 22 | *“Researchers should look into what specific things make people target them. Is it noises? Is it being very good in a subject? Is it because they know they are autistic etc.”*  *“Identification of risk factors that make autistic people particularly susceptible to certain kinds of abuse”* |
| 1. Are trauma/victim support services/approaches meeting the needs of autistic people, and what is helpful for recovery/creating safety? | 20 | *“Are support services (e.g., Women's Aid, Rape Crisis, etc.) able to meet the needs of autistic survivors? What support do autistic people need for recovery from trauma?”*  *“What has helped in recovery of CPTSD/PTSD”* |
| 1. How can trauma and victimisation be prevented and recognised early in autistic people? | 17 | *“How to help autistic people to recognise when they are being victimised.”*  *“Informing autistic people on how they should be treated and how to recognise when they are being mistreated.”* |
| 1. What is the impact of trauma and victimisation on autistic people in all areas of their lives across the lifespan? | 12 | *“The long-term effects of childhood bullying”*  *“What is the true impact (personally, mentally, socially, economically etc.) of trauma/ interpersonal violence?* |
| 1. How prevalent is victimisation and trauma for autistic people | 10 | *“How prevalent is interpersonal victimisation of autistic people? How many autistic people feel unsafe at home? At school? At work?”*  *“What percentage of autistic adults are subjected to domestic abuse, whether by intimate partners, parents, or housemates?”* |
| ***Priority eight: Education.*** | | |
| 1. How can educational institutions best support autistic individuals to reach their full potential (including into the future)? | 20 | *“How schools can support autistic kids appropriately giving good educational outcomes”*  *“How educational institutions can support autistic people.”* |
| 1. What does the most accessible and inclusive educational environment look like? | 10 | *“How can schools be made more suited to the needs of autistic pupils?”*  *“How can educational environments be made more accessible?”* |
| 1. How can we make teachers and other educational professionals more understanding about the needs of autistic individuals? | 7 | *“How can we improve teacher training?”*  *“What helps teachers to accommodate students?”* |
| 1. What would reduce non-attendance or dropping out of education? | 4 | *“Why is the dropout rate of autistic students at university so high?”*  *“Why is there a high rate of autistic children either not wanting to attend school or out of school?”* |
| 1. How can we support children, young people and families to advocate for their needs and ensure their voices are heard within education? | 2 | *“How can autistic children and teenagers have their voices heard with regard to their schooling?”*  *“How can we support children and families to advocate for their needs?”* |
| ***Priority nine: Sensory processing.*** | | |
| 1. What are the underlying mechanisms or causes of sensory processing differences and sensory overwhelm? | 9 | *“Are we experiencing sensory processing disorders because our senses aren't set up for a constant bombardment of social information in a modern world, extreme standards of dress emphasising image over comfort and practicality, endless noise pollution and artificial scents, etc.”*  *“What underlying biological issues cause sensory overload?”* |
| 1. How can sensory environments be adapted and improved? | 8 | *“The world we live in is so full of sensory input! If we have good research showing the absolute impact it has then it will help when designing schools, work places, services etc.”*  *“How can we design a society where sensory processing is taken seriously in public health to maximise autistic people's participation in society, to the benefit of everyone (we are a canary in the coal mine).”* |
| 1. How do autistic people's senses work and how is this different from neurotypicals? | 7 | *“How does autistic sensory processing work differently - structurally and functionally?”*  *“Are our senses heightened or are we more in tune with them”* |
| 1. How can we improve others' understanding of sensory processing? | 5 | *“How can we improve mental health teams, GP surgeries, and supporting organisation’s understanding of what sensory issues are”*  *“Sensory profile went through the roof at peri-menopause. More info for GPs on its significance”* |
| 1. What are the impacts of sensory processing difficulties/differences? | 4 | *“The impact of sensory issues on mental health.”*  *“I want research done into how big an impact the muddling of different sensations has on our physical health”* |
| 6= What coping strategies help autistic people cope with sensory processing difficulties? | 3 | *“I'd like to see research into what the best types of ear defenders are for those of us with noise sensitivity and how much of a difference it makes to our stress levels to be able to wear them when we need to.”*  *“How can less painful and overstimulating versions of helpful and necessary technology be developed e.g., screen filters, fridges and lighting that do not buzz painfully?”* |
| 6= How can positive sensory experiences be achieved and supported? | 3 | *“I would like to see research done on what stims and sensory adjustments and coping strategies making life the most comfortable and enjoyable (across ALL areas of life, from eating to having sex) for autists with different sensory profiles.”*  *“What is detrimental/beneficial to maintaining 'sensory harmony'”* |
| ***Priority ten: Life skills.*** | | |
| 1. How can autistic people be best supported with the skills needed for living independently and everyday life? | 24 | *“There is not enough support to help autistic adults learn and prioritise the life skills they need to live independently.”*  *“Could a system be developed to recognise and support autistic people to live well independently and provide appropriate life skills, support and advice, including to those who might function well in other areas of their lives?”* |
| 1. What support would help autistic people to manage money and their finances? | 5 | *“Assistance for financial planning”*  *“Looking after money”* |
| 1. How can autistic people be supported to self-advocate and understand their own needs, including recognising burnout? | 4 | *“How to be kind to yourself e.g. recognising difficulty with everyday tasks (that neurotypicals don't find taxing) is valid and needing to recover is not lazy”*  *“The educated understanding of needing a quiet home with ‘essential TIME OUT’.”* |
| 1. What role do executive functions play in living independently? | 2 | *“How disabling is executive dysfunction? To what extent is it behind social stresses, work struggles, and overall anxiety?”*  *“How to recognise hyperfocus and transition to the next task”* |

**B. “Other topics” mentioned with fewer than four comments, including number of comments (n) and example quotes.**

| **Topic** | **n** | **Example quote** |
| --- | --- | --- |
| Listening more to autistic people, including more participatory research | 4 | *“I think, for me, research needs to move away from being "about" autistic people and become more inclusive of and responsive to needs in a way that could lead to change in a real sense (not just in a research lab or research paper).”* |
| Crime | 3 | *“Autistic people in the criminal justice system and police custody.”* |
| Built environment accessibility | 3 | *“It's clear that the design of buildings and public spaces can massively impact the lives of autistic people positively and negatively and so more research into this area is definitely required.”* |
| Understanding heterogeneity | 3 | *“The broadness of autism, how different one autistic person can be from another in terms of capabilities.”* |
| Autistic thriving | 2 | *“Basically, how can we ensure that people like me are allowed to thrive and meet our potential, regardless of background or income?”* |
| Creativity and the arts | 1 | *“Autistic people in the arts.”* |
| Inertia | 1 | *“Autistic inertia and catatonia”* |
| Relationship with nature | 1 | *“The relationship between autistic people and the natural environment.”* |
